# Antibiotic Susceptibility Patterns and Prevalence of Streptococcus Agalactiae Rectovaginal Colonization Among Pregnant Women in Iran

**DOI:** 10.1055/s-0040-1710299

**Published:** 2020-06-19

**Authors:** Mina Dashtizade, Mohammad Reza Zolfaghari, Masoud Yousefi, Ali Nazari-Alam

**Affiliations:** 1 Department of Microbiology, Qom Branch, Islamic Azad University, Qom, Iran; 2 Birjand Infectious Diseases Research Center, Department of Microbiology, Faculty of Medicine, Birjand University of Medical Sciences, Birjand, Iran; 3 Department of Microbiology, Faculty of Medicine, Kashan University of Medical Sciences, Kashan, Iran

**Keywords:** *Streptococcus agalactiae*, pregnant women, antibiotic resistance, risk factors, polymerase chain reaction

## Abstract

**Objective** *Streptococcus agalactiae* is an important pathogen in neonates and pregnant women. Neonatal invasive infections due to *S. agalactiae* are life-threatening and preventive strategies for this challenge of human have become a concern. The aim of the present study was to determine the prevalence of rectovaginal colonization, related risk factors and antibiotic resistance pattern of *S. agalactiae* among pregnant women in Iran.

**Methods** The present study was performed on 240 pregnant women. Vaginal and rectal swabs were obtained from all of the women and then were transferred to the laboratory. The isolation and identification of *S. agalactiae* was performed by standard microbiological tests and polymerase chain reaction (PCR) assay. The antimicrobial susceptibility patterns of the isolates were determined by the Kirby-Bauer disk diffusion. Polymerase chain reaction was used to detect *ermB* and *mefA* genes in erythromycin-nonsusceptible isolates.

**Results** Out of 240 pregnant women, 16 cases (6.7%) were colonized by *S. agalactiae* . There is no significant association between demographic-obstetric factors and maternal *S. agalactiae* colonization in the pregnant women. Linezolid, vancomycin and ampicillin were the most effective antibiotics against *S. agalactiae* . The *ermB* gene was present in 6 (35.29%) *S. agalactiae* isolates. However, the *mefA* gene was not detected in any of the isolates.

**Conclusion** Given the relatively significant prevalence of *S. agalactiae* colonization in the pregnant women in the present study and the risk of serious neonatal infections, the screening of pregnant mothers for the bacteria seems necessary. Our findings highlight the importance of appropriate antibiotic prophylaxis during pregnancy for the prevention of early onset *S. agalactiae* -neonatal infection and comorbidity.

## Introduction

*Streptococcus agalactiae* (Group B *Streptococcus* [GBS]) is considered as the dominant pathogen in causing septicemia and meningitis in infants < 3 months old. Neonatal invasive infections due to *S. agalactiae* are life-threatening and preventive strategies for this challenge of human have become a concern. 1 2

As an important opportunistic human pathogen, GBS can be colonized in the rectovaginal area of women and subsequently transmitted to the neonates in the womb or during labor. The rate of GBS colonization among pregnant women varies with ethnic group, marital status, number of deliveries, geographic area and age. 3 4 It is noteworthy that ∼ between 10 and 30% of women during pregnancy are colonized with *S. agalactiae* in the vagina and 60% of their infants acquire the bacteria through the birth canal. 5 6 Identification of maternal GBS colonization during pregnancy is important for taking preventive measures to control neonatal diseases. 1 2

The Centers for Disease Control and Prevention (CDC), in order to reduce the incidence of neonatal GBS diseases, recommends the use of intrapartum antibiotic prophylaxis in pregnant women with rectovaginal colonization of GBS. However, the widespread adoption of intrapartum antibiotic prophylaxis for prevention of invasive early-onset GBS disease has led to an increase in concerns regarding the emergence of antibiotic resistance among GBS strains. So, the antibacterial susceptibility data of maternal colonizing GBS strains are essential to selective intrapartum antibiotic prophylaxis and minimize the emergence of bacterial resistance, which is causing increasing numbers of treatment failures. 7 8 9

Given the importance of universal screening of mothers for rectovaginal GBS colonization and achieving appropriate intrapartum antibiotic prophylaxis for all screen-positive women to prevent early-onset GBS-related diseases, the aim of the present study was to investigate the prevalence and related risk factors of GBS rectovaginal colonization in pregnant women as well as the antimicrobial susceptibility pattern of the isolates.

## Methods

### Study Population and Sampling Procedure

The present cross-sectional study was conducted among 240 pregnant women with gestational age of between 35–37 weeks referred to the Kashan Shahid Beheshti Hospital from January to September 2017. After receiving permission from the Ethics Committee of Kashan University of Medical Sciences (IR.KAUMS.PEC.1394.151), sociodemographic and clinical data were collected using a structured questionnaire.

Samples were taken using two sterile cotton swabs from the vaginal and rectal area according to the CDC and American College of Obstetricians and Gynecologists (ACOG) guidelines, 9 10 and inoculated directly into Todd-Hewitt broth (THB) (Merck & Co., Kenilworth, NJ, USA) supplemented with gentamicin (8 μg/ml) and nalidixic acid (15 μg/ml) (Sigma Aldrich, St. Louis, Missouri, USA), then were immediately transported to the microbiology laboratory within 2 hours of collection.

### Phenotypic Identification of Group B Streptococci

The broth media were incubated for between 18 and 24 hours at between 35–37°C and inoculated on 5% sheep blood agar (SBA) (Merck & Co., Kenilworth, NJ, USA) and incubated overnight in 5% CO2 atmosphere for between 18–24 hours. Finally, suspected GBS colonies (pink colonies, with narrow β-hemolysis) were identified by conventional microbiological and biochemical methods, including Gram stain, catalase test, bacitracin and sulfamethoxazole-trimethoprim (SXT) susceptibility tests, hippurate hydrolysis test (Mast Group Ltd, Bootle, UK), and Christie, Atkins, and Munch-Peterson (CAMP) test. 9 11

### PCR Confirmation of GBS Isolates

The PCR assays were used to confirm the diagnosis of GBS isolates by detecting the *dltS* target gene ( Table 1 ). Genomic DNA was extracted from pure cultures of the strains using High Pure PCR Template Preparation Kit (Roche, Basel, Switzerland) according to the instructions of the manufacturer. Polymerase chain reaction was conducted on the summation of all volumes consisting of 25 μL (12.5 μL of 2× Hot Star Taq Master Mix, 1 μL of the DNA template, 1 μL of each primer [50 pmol/μl] and 9.5 μL of ddH2O) using the Hot Star Taq Master Mix kit (SinaClon, Tehran, Iran). Settings for the reaction were as follows: initial denaturation step at 94°C for 5 minutes; 35 amplification cycles each for 30 seconds at 94°C, 30 1 minute at 55°C and 1 minute at 72°C. This was followed by an additional extension step of 10 minutes at 72°C. The PCR product of the *dltS* gene was electrophoresed on 1% agarose gel containing 1x RedSafe DNA stain (Intron Biotechnology, Seoul, South Korea).

**Table 1:** Target genes and their primers used in the present study

Primer	Sequence (5′-3′)	Products sizes (bp)	Annealing (°C)	Ref.
*dltS*	Fw- AGGAATACCAGGCGATGAAC Rv- TGCTCTAATTCTCCCCTTATGGC	952	55	(7)
*ermB*	Fw- CGACGAAACTGGCTAAAATA Rv- AATTGCTGAATCGAGACTTG	331	58	Present Study
*mefA*	Fw- GGTGTGCTAGTGGATCGTC Rv- GTAACCGCATTGAGAGCCG	188	53	Present Study

### Antibiotic Susceptibility Testing

The antibiotic resistance profile of the isolates was determined by the Kirby-Bauer disk-diffusion method on Muller-Hinton agar (MHA) (Merck & Co., Kenilworth, NJ, USA) with 5% sheep's blood, and the results were interpreted according to the Clinical and Laboratory Standards Institute (CLSI) guidelines. 12 The antimicrobial agents (Mast Group Ltd, Bootle, UK) tested in the present study included ampicillin (10 μg), vancomycin (30 μg), erythromycin (15 μg), clindamycin (2 μg), levofloxacin (5 μg), chloramphenicol (30 μg), cefepime (30 μg), and linezolid (30 μg). *Streptococcus pneumoniae* ATCC 49619 was used for quality control of antibiotic susceptibility testing.

### Detection of Erythromycin Resistance Genes

In the present study, due to the high rates of resistance to erythromycin in GBS, the mechanism of resistance to this antibiotic was studied with detection of *ermB* and *mefA* genes by PCR. The PCR was performed in the total volume of 25 μl (12.5 μl of 2x Hot Star Taq Master Mix, 1 μl of the DNA template, 1 μl of each primer [50 pmol/μl] and 9.5 μl of ddH2O) utilizing the Hot Star Taq Master Mix kit (SinaClon, Tehran, Iran). DNA amplification was performed in a thermocycler (Eppendorf, Hamburg, Germany) with an initial denaturation step at 95°C for 5 minutes, 30 amplification cycles each with 30 seconds at 95°C; 30 seconds at different temperatures for the various genes ( Table 1 ); and 40 seconds at 72°C, followed by an additional extension step of 7 minutes at 72°C. The amplified products were electrophoresed on 1.5% gel agarose containing 1x RedSafe DNA stain (Intron Biotechnology, Seoul, South Korea). Sequencing of amplicons was done by the Bioneer Company (Daejeon, South Korea). The BLAST program from the national center for biotechnology information (NCBI) Web site ( http://www.ncbi.nlm.nih.gov/BLAST ) was used to analyze the nucleotide sequences.

### Statistical Analysis

The data were analyzed with the Pearson chi-squared and the Fisher exact tests, using SPSS Statistics for Windows, Version 21.0 (IBM Corp. Armonk, NY, USA), to evaluate the statistical significance of associations between potential variables. P-values < 0.05 were considered to be significant.

## Results

In the present study, a total of 240 pregnant women (from 35 to 37 weeks of gestation) were enrolled. The mean age of the participants was 26.9 ± 4.41 years old, with the youngest being 16 and the oldest 45 years old.

### GBS Colonization and Related Risk Factors

The results indicated that 16 (6.7%) among the 240 pregnant women screened were colonized by *S. agalactiae* in their rectovaginal area. Of the 16 colonized patients, 8 had strains cultured only from vaginal swabs (50%), while 5 had strains isolated only from rectal swabs (31.25%) and another 3 had strains isolated simultaneously from both the vaginal and rectal swabs (18.75%). Overall, the GBS vaginal and rectal colonization rates were 4.58% and 3.33% respectively, while concomitant rectovaginal colonization rate was reported as 1.25%. The sociodemographic and pregnancy-related characteristics of the pregnant women and their relationship with maternal rectovaginal colonization of GBS are summarized in Tables 2 and 3 . Statistical analysis results showed that there was no significant association between demographic-obstetric factors and colonization of the maternal rectovaginal region with GBS.

**Table 2:** Association between sociodemographic factors and GBS colonization among pregnant women

Variables	Frequency n(%)	Culture positive n(%)	*p-value*
Age group (years old)			0.494
20 ≤	12 (5)	2 (16.7)	
21–30	116 (48.3)	6 (5.2)	
31–35	72 (30)	5 (6.9)	
36–45	40 (16.7)	3 (7.5)	
Education			0.929
Illiterate	53 (22.1)	3 (5.7)	
Pre-high school	35 (14.6)	2 (5.7)	
High school	93 (38.8)	6 (6.5)	
College	59 (24.6)	5 (8.5)	
Occupation			0.676
Housewife	218 (90.8)	15 (6.9)	
Employed	22 (9.2)	1 (4.5)	
Ethnicity groups			0.541
Iranian	214 (89.2)	15 (7)	
Afghan	26 (10.8)	1 (3.8)	

**Table 3:** Association between pregnancy-related characteristics and Group B *Streptococcus* colonization among pregnant women

Variables	Frequency n(%)	Culture positive n(%)	*p-value*
Gravidity			0.137
Primigravida	79 (32.9)	8 (10.1)	
Multigravida	161 (67.1)	8 (5)	
Type of delivery			0.251
Without delivery	77 (32.1)	8 (10.4)	
Vaginal	86 (35.8)	5 (5.8)	
Cesarean	77 (32.1)	3 (3.9)	
History of Abortion			0.155
Yes	67 (27.9)	2 (3)	
No	173 (72.1)	14 (8.1)	
Contraceptive methods			0.777
None	45 (18.8)	5 (11.1)	
Withdrawal	124 (51.7)	6 (4.8)	
Condom	51 (21.3)	4 (7.8)	
DMPA* injection	16 (6.7)	1 (6.3)	
OCP**	2 (0.8)	0 (0)	
IUD***	2 (0.8)	0 (0)	
Vaginal infection			0.752
Yes	99 (41.3)	6 (6.1)	
No	141 (58.8)	10 (7.1)	

Abbreviations: DMPA, depomedroxyprogesterone acetate; IUD, intrauterine device; OCP, oral contraceptive pills.

### Antibiotic Susceptibility

The results of antimicrobial susceptibility testing also showed that GBS isolates isolated from pregnant women was susceptible mainly to linezolid (100%), vancomycin (100%), and ampicillin (89.5%) ( Table 4 ). The intermediate antimicrobial resistance of GBS was 15.8% against erythromycin, 10.5% against ampicillin and levofloxacin, and 5.3% against clindamycin. It is noteworthy that the highest antibiotic resistance of GBS was related to erythromycin (73.7%).

**Table 4:** Antimicrobial susceptibility pattern of Group B *Streptococcus* isolates from pregnant women

Antibiotics	Susceptible (%)	Intermediate (%)	Resistant (%)
Erythromycin	2 (10.5)	3 (15.8)	14 (73.7)
Clindamycin	8 (42.1)	1 (5.3)	10 (52.6)
Ampicillin	17 (89.5)	2 (10.5)	0 (0)
Chloramphenicol	11 (57.9)	0 (0)	8 (42.1)
Levofloxacin	13 (68.4)	2 (10.5)	4 (21.1)
Cefepime	13 (68.4)	0 (0)	6 (31.6)
Linezolid	19 (100)	0 (0)	0 (0)
Vancomycin	19 (100)	0 (0)	0 (0)

### Prevalence of Erythromycin Resistance Genes

In the present study, the mechanism of resistance to erythromycin in the GBS isolates was studied with detection of *ermB* and *mefA* genes. The *ermB* gene was identified in 6 (35.29%) erythromycin nonsusceptible isolates. However, the *mefA* gene was not detected in any of the isolates.

## Discussion

In the last few decades, GBS has gained importance due to its implication in adverse obstetric outcomes, and its ability to cause serious neonatal infections. Epidemiological studies have revealed that GBS-colonized pregnant women are > 25 times more likely to deliver infants with early-onset GBS disease. 13 14

In the present study, the overall prevalence of GBS colonization among pregnant women was found to be 6.7%. This finding was slightly lower than many other reports in Iran 15 16 17 and other developing countries such as Ethiopia (7.2%), Turkey (8%), China (7.1%) and Korea (8.3%), 13 18 19 20 but higher than those reported in India (2.3%) and Taiwan (6.2%). 3 21 However, there are reports of higher rates of GBS colonization compared with our study from Tanzania (23%), Taiwan (21.8%) and Brazil (28.4%). 14 22 23 These disparities could be explained by the fact that rates of maternal GBS colonization during pregnancy varies in the worldwide population, possibly due to differences in the studied populations (in terms of age, ethnic group, socioeconomic status, sexual behavior and geographic areas), method of sample collection and the diagnostic techniques. It is noteworthy that in the present study the GBS vaginal and rectal colonization rates were 4.58% and 3.33%, respectively, while concomitant rectovaginal colonization rate was reported as 1.25%. This finding was almost similar with other studies 3 7 14 and reveals that multisite swabbing may be important in identifying GBS colonization.

Knowledge about the risk factors associated with GBS colonization during pregnancy can be important in reducing the incidence of maternal GBS infections and related neonatal morbidity and mortality. 24 25 The results of our study showed that there is no significant association between demographic-obstetric factors and maternal GBS colonization in pregnant women. Similar findings have been reported in studies conducted elsewhere. 24 26 27 28 However, in most other studies, the GBS colonization rate has been associated with some sociodemographic and pregnancy-related characteristics of the pregnant women. 3 5 14 16 This might be due to the small sample size in the present study. Finally, it was noteworthy in our study that primigravida women were more often associated with GBS colonization, though it was not statistically significant. Similar findings have been reported in studies from Ethiopia, Nigeria, Brazil and India. 5 24 25 29 30 However, in other studies, the GBS colonization rate was significantly higher in multigravida compared with primigravida women. 3 20 31 This difference may be due to geographical variation and shows that further studies are needed to confirm the correlation between gravidity and GBS colonization among pregnant women in different geographical locations.

Chemoprophylaxis remains the most effective means to prevent GBS maternal and neonatal infections. 9 13 The GBS strains isolated in the present study showed higher susceptibility to linezolid, vancomycin, and ampicillin. These results are consistent with the CDC clinical guidelines for the use of penicillin and ampicillin as the drugs of choice in prevention or treatment of GBS infections. 5 24 Furthermore, similar results were reported by other studies with high susceptibility rates of GBS strains to amoxicillin, linezolid and vancomycin. 7 32 33 It is noteworthy that clindamycin and erythromycin were recommended as antibiotic alternatives for penicillin-allergic women at high risk for anaphylaxis. However, recent reports had raised global concerns about increasing emergence of antimicrobial resistance to these antibiotics in GBS isolates. In the present study, the rates of resistance to erythromycin and clindamycin were reported as 73.7% and 52.6%, respectively. However, the erythromycin resistance rate among GBS isolates in the present study was relatively higher when compared with other studies in Iran, 34 35 36 but the high rates of resistance to erythromycin in GBS were reported in China (92.5% and 84.6%), Iraq (58.6%) and the USA (50.7%). 37 38 39 40 Furthermore, high resistance rates of GBS strains to clindamycin were reported from Iran (92.2%) and other countries such as China (55.7% and 87.5%), Iraq (45.6%) and Italy (32.20%). 19 37 39 41 It is noteworthy that the high rate of erythromycin and clindamycin resistance in GBS strongly supports the CDC recommendations for susceptibility testing of GBS isolates before initiating prophylaxis with erythromycin or clindamycin.

Finally, the results of the present study showed that the *ermB* gene was present in 35.29% of GBS erythromycin-nonsusceptible isolates. However, the *mefA* gene was not detected in any of the isolates. Similar findings have been reported in other studies, in which the methylation of target encoded by *ermB* genes was one of the commonest mechanisms of resistance to erythromycin in GBS isolates. 23 42 In addition, in some studies similar to our study, the *mefA* gene has not been identified in erythromycin-resistant GBS isolates. 23 43 This result implies that there are additional mechanisms involved with erythromycin resistance, which require further investigation.

## Conclusion

Given the relatively significant prevalence of *S. agalactiae* colonization in the pregnant women of the present study and the risk of serious neonatal infections, the screening of pregnant mothers for the bacteria seems necessary. Our findings highlight the importance of appropriate antibiotic prophylaxis during pregnancy for the prevention of early onset *S. agalactiae* -neonatal infection and comorbidity. It is noteworthy, considering the increasing concern about emergence of antimicrobial resistance to erythromycin and clindamycin as antibiotic alternatives in GBS isolates, that the susceptibility testing of the isolates before initiating prophylaxis with these antibiotics is recommended. Finally, further studies are needed to assess the correlation between different risk factors and maternal GBS colonization during pregnancy in various geographical locations.
